# Injectable Light-Responsive Hydrogel Dressing Promotes Diabetic Wound Healing by Enhancing Wound Angiogenesis and Inhibiting Inflammation

**DOI:** 10.3390/polym17050607

**Published:** 2025-02-25

**Authors:** Peifen Ma, Jianlong Da, Guanghui Zhao, Feiya Suo, Yan Li, Xiaochun Zhou, Yao Li, Yiheng Han, Mingyang Zou, Xinman Dou

**Affiliations:** 1 The Second Hospital & Clinical Medical School, Lanzhou University, Lanzhou 730000, China; ldyy_mapf@lzu.edu.cn (P.M.); dajl20@lzu.edu.cn (J.D.); suofy20@lzu.edu.cn (F.S.); liyanhl@lzu.edu.cn (Y.L.); zhouxiaochun89@163.com (X.Z.); liyao21@lzu.edu.cn (Y.L.); 320220906461@lzu.edu.cn (Y.H.); zoumy2021@lzu.edu.cn (M.Z.); 2 State Key Laboratory of Applied Organic Chemistry, College of Chemistry and Chemical Engineering, Lanzhou University, Lanzhou 730000, China; zhaogh@lzu.edu.cn

**Keywords:** injectable hydrogel, light-responsive hydrogel dressing, diabetic wounds, antibacterial, anti-inflammatory, angiogenesis

## Abstract

Diabetic wounds are therapeutically challenging because of the complex and adverse microenvironment that impedes healing. Unlike conventional wound dressings, hydrogels provide antibacterial, anti-inflammatory, and repair-promoting functions. In this study, we developed a light-responsive and injectable chitosan methacryloyl (CSMA) hydrogel, incorporating soy isoflavones (SIs) and gold nanoparticles (AuNPs). Transmission electron microscopy (TEM), Fourier transform infrared (FTIR) spectroscopy, and proton nuclear magnetic resonance (1H NMR) spectroscopy analyses confirmed the successful synthesis of the CSMA/SI/AuNP hydrogels. In vitro experiments demonstrated that this hydrogel exhibited exceptional biocompatibility and enhanced the migration of human umbilical vein endothelial cells (*p* < 0.05), thereby underscoring its potential for promoting angiogenesis. In vivo studies have indicated that hydrogels significantly enhance the rate of wound healing (*p* < 0.001). Moreover, they facilitate angiogenesis (*p* < 0.01) and diminish the inflammatory response at the wound site (*p* < 0.05). Additionally, hydrogels promote collagen deposition and the regeneration of skin appendages. These findings substantiate the hydrogel’s therapeutic potential for diabetic wound care, highlighting its promise for regenerative medicine. CSMA/SI/AuNP represents a significant advancement in diabetic wound treatment, addressing key challenges in wound healing by offering a multifaceted therapeutic approach with broad clinical implications for enhancing patient outcomes in chronic wound management.

## 1. Introduction

Diabetic wounds are a prevalent and serious complication of diabetes, with an incidence rate of up to 25% [1]. The microenvironment of diabetic wounds is characterized by hypoxia, hyperglycemia, excessive reactive oxygen species (ROS), and bacterial infection, impairing cellular functions and disrupting normal gene expression. These factors contribute to the prolonged or inhibited progression of essential processes, such as angiogenesis, collagen deposition, and tissue remodeling, ultimately resulting in chronic non-healing wounds. Consequently, patients often experience extended hospitalization with an increased risk of amputation and mortality. Currently, wound dressings are the primary treatment for diabetic wounds [2]. However, traditional dressings typically possess limited functionality; they are inadequate for preventing infection and fail to mitigate local inflammation or improve the wound microenvironment. Moreover, repeated changes in conventional dressings may induce secondary injury to the newly formed granulation tissue [3]. Hydrogels have emerged as an ideal platform for promoting the healing of diabetic wounds, owing to their antibacterial properties, ease of application, moisture-retentive capabilities, and ability to regulate inflammation [4,5]. Hydrogels with superior biocompatibility, robust tissue adhesion, and self-repairing properties are considered ideal for clinical applications. Current hydrogel dressings available in the market, such as Hydrosorb^®^ by HARTMANN and 3M Tegaderm, facilitate wound healing primarily through their moisturizing and water-absorbing properties, while also physically isolating the wound from the external environment. However, these products typically operate via a single mechanism and do not directly accelerate the wound healing process. Additionally, many commercial hydrogel dressings are pre-formed and thus cannot accommodate wounds with complex shapes and varying depths. Injectable hydrogels, on the other hand, enable precise filling of irregularly shaped wounds [6]. Compared to pre-structured biological dressings, injectable hydrogels offer a broader range of applications and are particularly suitable for treating complex wounds [7]. The transition of hydrogels from an injectable liquid state to a solid state with adequate mechanical strength, triggered by stimuli such as light, temperature, pH, and local reactive oxygen species (ROS) levels, is essential for their application in complex wound healing [8,9,10]. Among these, light-responsive hydrogels are particularly advantageous due to their cost-effectiveness, non-contact nature, and suitability for dynamic cellular microenvironments and controlled drug release [11].

Photoinitiated materials are predominantly synthesized through chemical modification, with commonly selected substrates including chitosan, hyaluronic acid, and multi-arm poly(ethylene glycol) (PEG), due to their superior biocompatibility [8,12,13]. Chitosan (CS), a biopolymer derived from chitin deacetylation, is primarily composed of glucosamine and N-acetylglucosamine. It exhibits inherent antibacterial activity and excellent biocompatibility and biodegradability, making it widely used in tissue engineering [14]. Chitosan (CS) exhibits multiple degradation pathways, including photodegradation, oxidative degradation, and enzymatic degradation, with the predominant mode in physiological environments being enzymatic degradation [15]. Lysosomes play a crucial role in the enzymatic degradation process. Chitosan contains N-acetylglucosamine residues, which are recognized and degraded by lysosomal enzymes, resulting in the hydrolysis of chitosan into oligosaccharides of varying lengths composed of glucosamine and N-acetylglucosamine residues [16]. Additionally, chitosan can be hydrolyzed by over 30 different enzymes, including glucoamylase, protease, lipase, cellulase, and pectinase [17]. These hydrolytic enzymes exhibit high specificity and, in conjunction with lysosomal activity, facilitate the safe and efficient degradation of chitosan in vivo. Despite many advantages, CS has several intrinsic limitations, including solubility in weakly acidic solutions, along with poor mechanical strength when used in isolation. These shortcomings significantly restrict its clinical applicability. Consequently, modifying CS to enhance its mechanical and functional properties has become an important area of research. One promising approach involves grafting methacrylic acid (MA) onto CS, resulting in the formation of a chitosan methacryloyl (CSMA) copolymer, which possesses both injectability and photoresponsive characteristics. Recent studies have demonstrated that CSMA exhibits superior antibacterial activity and enhanced mechanical strength compared with CS. Furthermore, by incorporating additional bioactive agents, CSMA can be endowed with additional properties, including antioxidant and anti-inflammatory effects [18,19,20]. The free radical photoinitiator lithium phenyl-2,4,6-trimethylbenzoylphosphonate (LAP) has garnered significant interest due to its favorable properties. It demonstrates high water solubility, excellent biocompatibility, and effective cell encapsulation when utilized at low concentrations and under long light wavelengths (365–405 nm) [21]. Upon irradiation of a mixture containing LAP and CSMA with light of the appropriate wavelength, the LAP molecules in the mixture absorb the light energy, leading to their dissociation into two free radicals. These free radicals subsequently attack the carbon–carbon double bond (C=C) of CSMA, thereby initiating the photopolymerization reaction of the modified polysaccharide. This process culminates in the formation of three-dimensional polymers through the crosslinking of CSMA polymer chains [22].

Estrogen is intimately involved in the regulation of gene expression related to tissue repair and plays a pivotal role in the healing of diabetic wounds. Both local and systemic estrogen administration have been shown to enhance wound healing under diabetic conditions [23]. A study conducted by Yan et al. [24] demonstrated that estrogen treatment of skin wounds in diabetic mice significantly accelerated angiogenesis and collagen deposition, leading to faster wound closure than in the control group. These findings underscore the therapeutic potential of estrogen in the management of diabetic wounds. However, concerns about the safety of systemic estrogen therapy may limit its widespread clinical use [25]. Isoflavones, which are natural compounds predominantly extracted from soybeans and other plants, have several advantages over estrogen, including ease of production and milder biological effects, thus positioning them as suitable alternatives. Among various isoflavones, soy isoflavones are the most extensively studied. One study utilizing the active ingredient daidzein to treat diabetic wounds proposed that its therapeutic effect may be mediated through the dose-dependent inhibition of the FosO1/iNOS signaling pathway, which subsequently promotes wound healing [26].

Bacterial infection is a hallmark of diabetic wounds and remains a significant challenge that conventional dressings fail to adequately address. Although the use of chemical antimicrobial agents remains the standard clinical approach, the growing issue of antimicrobial resistance associated with these agents remains a major concern. In this context, metal nanoparticles have emerged as promising alternatives owing to their unique chemical properties, sizes, and shapes. These nanoparticles can exert bactericidal effects through direct interactions with bacterial cell walls rather than via chemical binding, thus reducing the likelihood of bacterial resistance. Recently, gold nanoparticles (AuNPs) have attracted considerable attention due to their ability to kill bacteria through mechanisms such as reactive oxygen species (ROS)-mediated DNA damage. This prevents membrane damage, disruption of cell walls, and structural interference, making AuNPs a promising tool in combating bacterial infections. They exhibit anti-inflammatory activity by modulating oxidative stress and inducing macrophage polarization [27,28]. Studies have demonstrated that AuNPs possess significant antioxidant properties because they can effectively scavenge free radicals. In vitro assays further revealed that AuNPs inhibited the activity of α-amylase and α-glucosidase, suggesting their potential antidiabetic effects. Furthermore, in vivo studies have shown that AuNPs significantly enhance the expression of NANOG and CD4 proteins in wound tissues, thereby promoting tissue repair and regeneration [29,30].

In this study, chitosan methacryloyl (CSMA), a biomaterial incorporating soy isoflavones (SI) and gold nanoparticles (AuNPs), with photoinitiation properties, was used as the base material, to exploit their respective biological advantages. The efficacy of CSMA/SI/AuNP hydrogel was evaluated by cellular and animal model experiments. The results indicated that the composite hydrogel exhibits significant antibacterial activity and immune modulation, promotes angiogenesis, and accelerates the healing of diabetic wounds. This study provides valuable experimental evidence that supports the development of novel hydrogels for the treatment of diabetic wounds.

## 2. Materials and Methods

### 2.1. Preparation of Methacryloylated Chitosan/Soy Isoflavone/Gold Nanoparticle (CSMA/SI/AuNP) Photoinitiated Hydrogel

Materials: chitosan (Macklin, Osaka, Japan, CAS: 9012-76-4), acetic acid (FUCHEN, Osaka, Japan, CAS: 64-19-7), methacrylic anhydride (Aladdin, London, UK, CAS: 760-93-0), sodium hydroxide (Aladdin, CAS: 1310-73-2), tetrachloroauric acid tetrahydrate (HUSHI, Tokyo, Japan, CAS: 16903-35-8), potassium carbonate (KESHI, Brussels, Belgium, CAS: 584-08-7), tannic acid (Aladdin, CAS: 1401-55-4), trisodium citrate (KESHI, CAS: 6132-04-3), soy isoflavones (Chemical BOOK, Beijing, China, CAS: 574-12-9), and photoinitiator LAP (FUCHEN, CAS: 64-19-7).

#### 2.1.1. Synthesis of Chitosan Methacryloyl (CSMA)

A predetermined amount of chitosan powder with a 50% degree of deacetylation was dissolved in a 1% acetic acid solution to prepare a 1% chitosan acetic acid solution. This solution was transferred to a round-bottom flask, to which methacrylic anhydride was added dropwise, stirring at an amino-to-anhydride molar ratio of 2:3. The reaction was carried out under reflux at 60 °C for 8 h. Following the reaction, the pH of the solution was adjusted to approximately 7.0, using 1 M sodium hydroxide solution. The mixture was subsequently dialyzed in ultrapure water using a 1000D molecular weight cutoff dialysis membrane for four days, with water exchanges performed twice daily. The dialysate was lyophilized to obtain chitosan methacryloyl (CSMA).

#### 2.1.2. Synthesis of Gold Nanoparticles (AuNPs)

Tetrachloroauric acid tetrahydrate (HAuCl_4_·4H_2_O) was dissolved in ultrapure water to prepare a 1% gold chloride solution. To this solution, 20 mL of ultrapure water, 0.56 mL of 1% trisodium citrate solution, 0.3 mL of 1% tannic acid solution, 0.3 mL of 1% potassium carbonate solution, and 0.5 mL of 1% HAuCl_4_ solution were sequentially added while stirring at room temperature. The final solution was adjusted to a total volume of 50 mL using ultrapure water, stirring rapidly until the solution exhibited no visible color change. The resulting colloidal solution of AuNPs was used for subsequent applications.

#### 2.1.3. Preparation of CSMA/SI/AuNP Photoinitiated Hydrogel

An aqueous solution of soy isoflavones was prepared at the desired concentration using ultrapure water. Lyophilized CSMA was added to the solution to achieve a final CSMA concentration of 20 mg/mL. The photoinitiator LAP was incorporated at a concentration of 10 mg/mL, and the mixture was stirred until the CSMA dissolved completely. Subsequently, the prepared AuNP solution was added to the mixture at a 1:10 ratio and mixed thoroughly. The final mixture was poured into molds and exposed to ultraviolet (UV) light at a wavelength of 365 nm and an intensity of 4 mW/cm^2^ for 30 s to induce gelation, yielding the CSMA/SI/AuNP-photoinitiated hydrogel.

### 2.2. Characterization of Hydrogel Properties

#### 2.2.1. Characterization of Gold Nanoparticle Size

To characterize the size of the gold nanoparticles, a 20 μL aliquot of the AuNPs solution was applied onto a 200-mesh carbon-coated copper grid. After allowing the solution to stabilize, excess liquid was removed using filter paper, and the grid was air-dried. The size and morphology of the AuNPs were analyzed by transmission electron microscopy (TEM; Hitachi HT7800, Tokyo, Japan).

#### 2.2.2. Fourier Transform Infrared (FT-IR) Spectroscopy

The freeze-dried samples were analyzed by Fourier transform infrared spectroscopy (FT-IR) using a FT-IR spectrometer (Thermo Scientific Nicolet iS5 FT-IR Spectrometer, Waltham, MA, USA). The spectra were recorded within the wavenumber range of 1000–1800 cm^−1^, with a resolution of 2 cm^−1^, to evaluate the chemical functional groups and structural characteristics of the materials.

#### 2.2.3. Nuclear Magnetic Hydrogen Spectroscopy Analysis

A 5 mg sample of the synthesized chitosan methacrylate (CSMA) was accurately weighed and dissolved in 0.55 mL of deuterium oxide (D2O). The solution was then transferred into an NMR tube using a syringe and subsequently analyzed by proton nuclear magnetic resonance (1H NMR) spectroscopy utilizing a Bruker AVANCE III 400 MHz NMR spectrometer (Billerica, MA, USA). The degree of substitution was calculated as follows:

(1) $$DS=\frac{A_{H(5.4\&5.6)}/2}{A_{H\left(2.5-4.1\right)}/6}\times 90\%.$$

#### 2.2.4. Gelation Behavior of the Hydrogel and Rheological Characterization

The CSMA/SI/AuNP solution was divided into two equal portions and placed in transparent containers labeled A and B. Container A was exposed to UV light for 30 s, whereas container B remained untreated. The gelation process was observed by tilting the containers to monitor gel formation. The dynamic rheological characterization of CSMA/SI/AuNPs hydrogels was conducted at room temperature using a NETZSCH Kinexus Lab+ rheometer (Selb, Germany). We measured the changes in storage modulus (G′) and loss modulus (G′′) of the hydrogels before and after 30 s of UV illumination, applying a strain of 0.5% at 25 °C. The frequency range for these measurements was set between 0.1 and 100 rad s^−1^.

#### 2.2.5. Self-Healing Performance

The CSMA/SI/AuNP hydrogel was initially prepared in solution form and injected into a mold, followed by UV irradiation at 365 nm for 30 s at an intensity of 4 mW/cm^2^. After demolding, the hydrogels were sectioned into two equal parts and subsequently separated. One segment was stained with Rhodamine B (Aladdin, CAS: 81-88-9) to facilitate visualization, and the two pieces were gently pressed together. Following a 5 min incubation at room temperature, the two segments were reassembled and self-healing was observed.

#### 2.2.6. Antibacterial Performance

The antimicrobial efficacy of CSMA/SI/AuNP hydrogels was evaluated by agar diffusion method. Holes were made in agar plates, and different groups of hydrogels (group 1 CSMA, group 2 CSMA/SI/AuNP, group 3 CSMA/SI, and group 4 CSMA/AuNP) were injected into the molds. After initiation by UV irradiation, the gelatinized hydrogels of the above groups were placed in the wells and fixed in agar. The groups were distributed axially in the agar plate. The concentration of the bacterial solution was adjusted so that its OD value at 600 nm of the enzyme marker was 0.101–0.103, i.e., 108 CFU/mL. Staphylococcus aureus and Escherichia coli were inoculated in the plates, respectively. The antimicrobial activity of each hydrogel was determined by measuring the size of the zone of inhibition around the hydrogel.

### 2.3. In Vitro Experimental Procedures

#### 2.3.1. Preparation of Hydrogel Extracts

The CSMA/SI/AuNP hydrogels were fabricated into solid discs with a diameter of 1.5 cm. Sterilization was achieved by exposing both sides of the hydrogel to UV radiation. After the hydrogels were weighed, they were immersed in complete RPMI-1640 culture medium at a concentration of 0.2 g/mL to extract the bioactive components. The hydrogel extracts were incubated at 37 °C for 24 h, after which they were collected in sterile containers for subsequent use.

#### 2.3.2. Cytotoxicity Assay

The L929 cells were enzymatically dissociated, centrifuged, resuspended, and seeded at a density of 3 × 10^4^ cells/well in a 6-well plate. The cells were cultured at 37 °C in a humidified atmosphere containing 50% CO_2_ for 24 h. In the experimental groups, 500 µL of 100% hydrogel extract was added to Group 1, 300 µL of 100% hydrogel extract to Group 2, and 500 µL of 50% hydrogel extract to Group 3. The control group did not receive any treatment. After a 24 h incubation, the culture medium was aspirated, and the cells were washed twice with phosphate-buffered saline (PBS). A calcein-AM/propidium iodide (PI) Live/Dead Cell Staining Kit (Solarbio, Beijing, China, CA1630) was equilibrated at room temperature prior to use. A 5 µL aliquot of calcein-AM and 15 µL of PI were added to 5 mL of 1× Assay buffer, ensuring that the final concentrations of calcein-AM and PI were 2 mM and 4.5 mM, respectively; 1 mL of the working solution was then added to each well, and the cells were incubated at 37 °C in the dark for 30 min. Fluorescence microscopy (Olympus, Tokyo, Japan, CKX53) was used to observe cell viability under an excitation wavelength of 490 ± 10 nm.

#### 2.3.3. Cell Proliferation Assay

Human umbilical vein endothelial cells (HuVECs) and murine fibroblasts (L929) were cultured in RPMI-1640 medium (Gibco, Waltham, MA, USA, C11875500BT) supplemented with 10% fetal bovine serum (Vazyme, Nanjing, China, F101-01). The experiment included five groups: hydrogel extracts of 100%, 75%, 50%, and 25%, and control. After enzymatic dissociation, centrifugation, and resuspension, the cells were seeded in 96-well plates at a density of 5 × 10^3^ cells/well, with five replicates per group. The cells were incubated at 37 °C in a 50% CO_2_ atmosphere for 24 h. After the incubation period, the medium was discarded, and 100 µL of fresh complete culture medium was added to each well. In the experimental groups, 50 µL of hydrogel extract was added at the specified concentrations, while the control group received no treatment. The cells were cultured for 24 h and 72 h. After incubation, the medium was removed, and each well was treated with 100 µL of 10% CCK-8 solution (Biosharp, Hefei, China, BS350B). Following a 1 h incubation at 37 °C and 50% CO_2_, the absorbance at 450 nm was measured using a microplate reader (EPOCH BioTek, Dover, MA, USA) to assess cell viability.

#### 2.3.4. Cell Migration Assay

The HuVECs were enzymatically dissociated, centrifuged, resuspended, and seeded at a density of 3 × 10^4^ cells/well in a 6-well plate. Once cell confluence exceeded 80%, a linear scratch was made across the monolayer using a 200 µL pipette tip. The medium was aspirated, and the cells were washed three times with phosphate-buffered saline (PBS). Fresh RPMI-1640 medium containing 2% fetal bovine serum (FBS) was added (2 mL per well). Migration was monitored using an inverted microscope, and images of the same region of interest were captured at baseline (0 h). For the experimental group, 300 µL of 100% hydrogel extract was added, while the control group received no treatment. After thorough mixing, the 6-well plate was returned to the incubator. Images were taken at 12 and 24 h, and the wound areas were quantified using the ImageJ software (ImageJ 1.51j8). The healing rate was calculated as follows:

(2) $$Healing\;Rate=\frac{Scratch\;Area\;at\;0h-Scratch\;Area\;at\;12h\;or\;24h}{Scratch\;Area\;at\;0h}\times 100\%.$$

### 2.4. In Vivo Experiments

Eight female DB/DB mice weighing between 35 and 42 g were obtained from Cavens Lab Animal Co., Ltd. (Changzhou, China). The DB/DB mice are leptin-deficient models produced through inbreeding. The db gene, a recessive mutation located on chromosome 4, induces obesity accompanied by diabetes, with homozygous individuals exhibiting pronounced hyperglycemia. The mice were randomly divided into two groups: the CSMA/SI/AuNP group and the blank control group. All animal experiments were approved by the Medical Ethics Committee of the Second Hospital of Lanzhou University (Approval No. D2024-844), in accordance with the ethical guidelines for animal research.

#### 2.4.1. Wound Healing Experiment

Under general anesthesia induced by an intraperitoneal injection of 3% pentobarbital sodium (50 mg/kg), the dorsal skin of the mice was shaved, and a full-thickness wound (diameter, 0.5 cm) was created on the neck. The wound area was disinfected, and the CSMA/SI/AuNP hydrogel (0.5 mL) was applied to the wound, followed by UV irradiation for 30 s. In the control group, an equivalent volume of saline was applied, and sterile dressings were secured. Photographs were taken on days 3, 5, and 7, and the wound area was measured using the ImageJ software. The wound healing rate was calculated as follows:

(3) $$Wound\;Healing\;Rate=\frac{Initial\;Wound\;Area-Wound\;Area\;on\;Day\;X}{Initial\;Wound\;Area}\times 100\%.$$

On day 14, the mice were humanely euthanized and tissue samples from the wound site were harvested and fixed in 10% formalin for further analysis.

#### 2.4.2. Histological Analysis

The harvested tissue samples were dehydrated using a graded ethanol series and embedded in paraffin. Cross-sections (3 µm thickness) were treated with Hematoxylin and Eosin (H&E) stain and Masson’s Trichrome stain. The tissue morphology and structural integrity were examined under a light microscope and photographed.

#### 2.4.3. Immunohistochemical Analysis

The paraffin-embedded tissue sections were deparaffinized and rehydrated, followed by antigen retrieval using MTris-EDTA buffer under high-pressure conditions (125 °C, 103 kPa). Endogenous peroxidase activity was blocked using 3% hydrogen peroxide in a humidified chamber. Sections were incubated overnight at 4 °C with primary antibodies targeting CD31 (Bioswamp PAB33199, Osaka, Japan), α-SMA (Bioswamp PAB30319), and IL-10 (Bioswamp PAB40480). After warming to room temperature, secondary antibody incubation was performed at 37 °C for 60 min. The sections were then stained with DAB and Hematoxylin, dehydrated, and mounted. Microscopic images were acquired, and staining intensities of CD31, α-SMA, and IL-10 were quantified using ImageJ software to assess angiogenesis and inflammatory responses.

### 2.5. Statistical Analysis

All experimental data collected were analyzed by one-way analysis of variance (ANOVA). *p*-values less than 0.05 were considered statistically significant (* *p* < 0.05, ** *p* < 0.01, *** *p* < 0.001, **** *p* < 0.0001).

## 3. Results and Discussion

### 3.1. Material Characterization

#### 3.1.1. Morphology and Size of AuNPs

**Figure 1:**
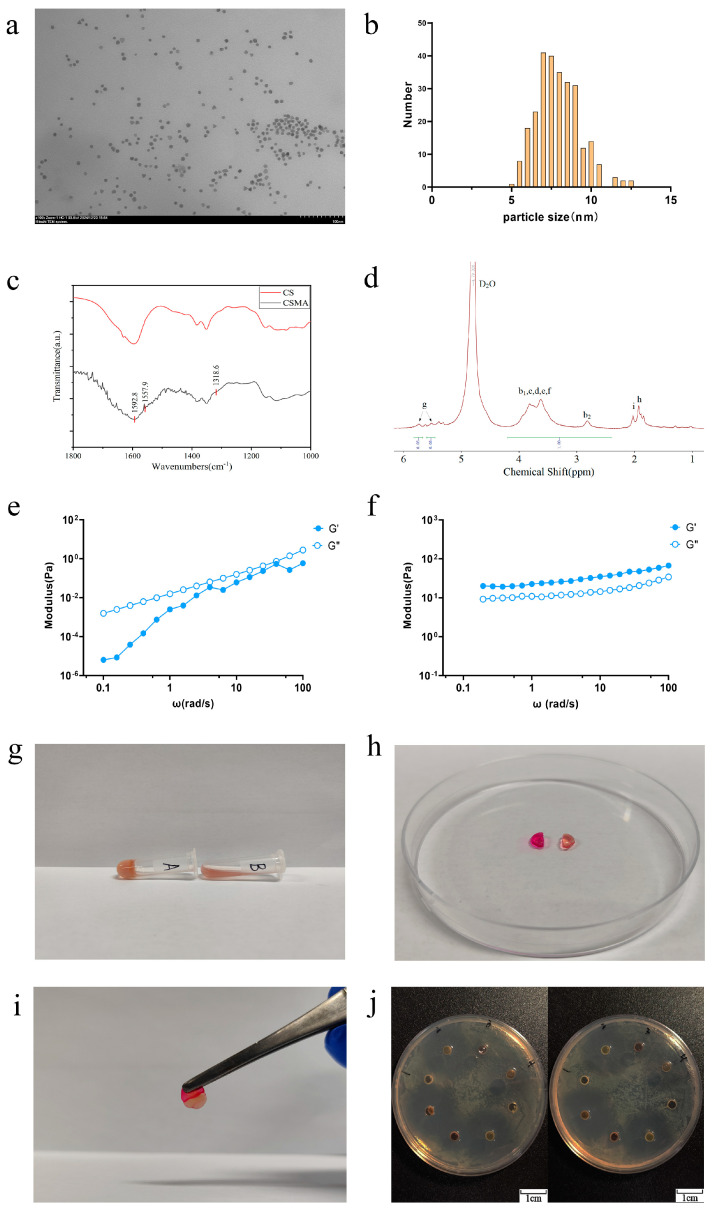
Characterization and antimicrobial properties of CSMA/SI/AuNP. (**a**) TEM images of AuNPs solution; (**b**) AuNP particle size distribution; (**c**) infrared spectrograms of CSMA and CS; (**d**) nuclear magnetic hydrogen spectroscopy analysis of CSMA. Peaks labeled b, c, d, and f represent the cyclic proton signals of the monosaccharides glucosamine and acetylglucose. Peak labeled g represents the signals from two distinct vinyl proton peaks. Peak labeled h represents the methyl signal of a methacrylic anhydride residue; (**e**) rheological characteristics before UV irradiation; (**f**) rheological characteristics after UV irradiation; (**g**) tilt placement of CSMA/SI/AuNP hydrogels after rapid gelation; (**h**) CSMA/SI/AuNP hydrogels were separated and stained after gelation; (**i**) the assembled hydrogels were left to stand for 5 min and then lifted up using tweezers; (**j**) Antibiotic Disk Diffusion Test of CSMA/SI/AuNP with Staphylococcus aureus on the left and *E. coli* on the right.

As evidenced by the transmission electron microscopy (TEM) images in Figure 1a, the synthesized AuNPs exhibited a uniform spherical morphology with particle sizes ranging from 7 to 10 nm (Figure 1b). The tannic acid–sodium citrate reduction method was employed to achieve this size distribution. An increase in tannic acid concentration led to a more homogeneous nanoparticle dispersion, consistent with previously reported findings [31].

#### 3.1.2. Infrared Spectroscopy of CSMA

The infrared spectra of chitosan and CHIMA are shown in Figure 1c. The key absorption peaks observed at 1592.8 cm^−1^, 1557.9 cm^−1^, and 1318.6 cm^−1^ correspond to the amide I (C=O stretching), amide II (N-H bending and C-N stretching), and amide III bands, respectively. These findings confirmed the successful incorporation of the maleic anhydride (MA) group into the chitosan backbone, thereby verifying the successful synthesis of CSMA.

#### 3.1.3. Nuclear Magnetic Hydrogen Spectroscopy Analysis of CSMA

CSMA was obtained by the amidation reaction of MA with the amino group in CS. Figure 1d shows the NMR hydrogen spectrum of CSMA. As shown, the chemical shifts at 2.5–4.0 ppm are the cyclic proton peaks of monosaccharide amino glucose and monosaccharide acetyl glucose (b, c, d, f). The chemical shift of Hb_2_ of monosaccharide glucosamine is at 2.9 ppm and that of Hb_1_ of monosaccharide acetyl glucosamine is at 3.0–4.0 ppm. There are two distinct signal peaks at 5.4 and 5.6 ppm for vinyl proton peaks (g), and a methyl peak of methacrylic anhydride residue at 1.8 ppm (h). The vinyl proton peak and methyl peak, which are not present in CS, appeared in Figure 1d, consistent with the structural formula of CSMA. It was verified that MA was grafted onto the molecular chain of chitosan and CSMA was successfully prepared. According to the literature [32], the degree of substitution (DS) of MA can be calculated based on the NMR hydrogen spectra. The degree of substitution of MA, which is also known as the degree of double-bond substitution, was calculated from the integral areas of the b_1_, b_2_, c, d, and f peaks in the hydrogen spectra in comparison with the integral area of the g peak. The double bond substitution of CSMA was calculated to be 27%.

### 3.2. Rheological Characterization and Gelation 

The rheological characteristics of the CSMA/SI/AuNP hydrogels, both prior to and following UV irradiation, are illustrated in Figure 1e,f. At a temperature of 25 °C and under a strain of 0.5%, the loss modulus (G″) exceeds the storage modulus (G′) before UV irradiation, indicating that the material is in a predominantly liquid state with inherent fluidity. In contrast, after 30 s of UV exposure, this relationship is inverted, with G′ surpassing G″, signifying a transition of the material to a more solid-like state. These observations confirm that the hydrogel can be effectively phototriggered by UV irradiation, leading to a shift from a fluidic to a solid hydrogel form, thereby substantiating its photoinitiated gelation properties.

Figure 1g illustrates the gelation process of the CSMA/SI/AuNP hydrogels. Upon exposure to UV light (10 mW/cm^2^ for 30 s), the hydrogel transitioned from a liquid to a solid gel and remained stable even when placed in an inclined position. Prolonged high-intensity UV exposure accelerates cellular senescence and induces skin damage [33]. Therefore, the initiation conditions for UV-sensitive materials require careful consideration. In this study, the CSMA/SI/AuNP hydrogel underwent gelation within a relatively short exposure time (30 s) under low light intensity (10 mW/cm^2^), thereby mitigating potential skin damage. Previous reports demonstrated that such exposure parameters do not result in significant skin cell damage, further supporting the hydrogel’s safety profile [34].

Hydrogels formed by dynamic crosslinking typically exhibit good self-healing properties and injectability. However, they often suffer from inadequate mechanical strength and lack sufficient structural support, which limits their ability to meet the elastic modulus requirements of various tissue types. As a light-responsive prepolymer [35], CSMA can facilitate the formation of a more compact internal structure within a hydrogel, thereby improving the elastic modulus and overall mechanical performance. In this study, we successfully synthesized chitosan methacrylate (CSMA) using a simplified one-step method, which enhanced its water solubility and significantly improved the hydrogel’s ability to address various types of wounds.

### 3.3. Self-Healing Capacity of CSMA/SI/AuNP Hydrogels

Figure 1h,i demonstrate that after 30 s of UV exposure and methyl orange staining, the CSMA/SI/AuNP hydrogel, when divided into two pieces and left to heal, successfully reattached and could be lifted without rupture, indicating its ability of self-healing and mechanical strength. This observation confirms the self-healing ability of the hydrogel.

### 3.4. Antibacterial Properties of CSMA/SI/AuNP Hydrogels

Recent studies have highlighted that the antibacterial efficacy of nanoparticles is influenced not only by their shape, surface-active agents, and reducing agents, but also by their size. Smaller AuNPs exhibited superior antibacterial activity [36]. As illustrated in Figure 1j, when the bacterial colony formation was assessed, the CSMA hydrogel displayed negligible antibacterial activity, possibly because of the relatively low concentration used in the experiment. Conversely, the CSMA/SI hydrogel, which did not contain any additional antibacterial agents, exhibited a zone of inhibition, suggesting some degree of antimicrobial activity. On the contrary, CSMA/SI hydrogel without any additional antimicrobial agent showed zones of inhibition, indicating some degree of antimicrobial activity. We also observed that CSMA/SI hydrogel inhibited *S. aureus* more than *E. coli*. This effect may be attributed to the presence of soy isoflavones, which have been shown to inhibit the growth of Staphylococcus aureus, Bacillus subtilis, Candida albicans, and Aspergillus oryzae at concentrations as low as 1%. However, no inhibition was observed against Escherichia coli or yeast strains [37]. The CSMA/AuNP hydrogel exhibited enhanced antibacterial properties against both organisms, confirming the well-established antibacterial effects of small AuNPs. Furthermore, the CSMA/SI/AuNP hydrogel demonstrated a larger zone of inhibition compared to CSMA, highlighting its superior antibacterial activity. These findings confirmed the antibacterial properties of the CSMA/SI/AuNP hydrogels.

### 3.5. In Vitro Cytotoxicity of CSMA/SI/AuNP Hydrogels

**Figure 2:**
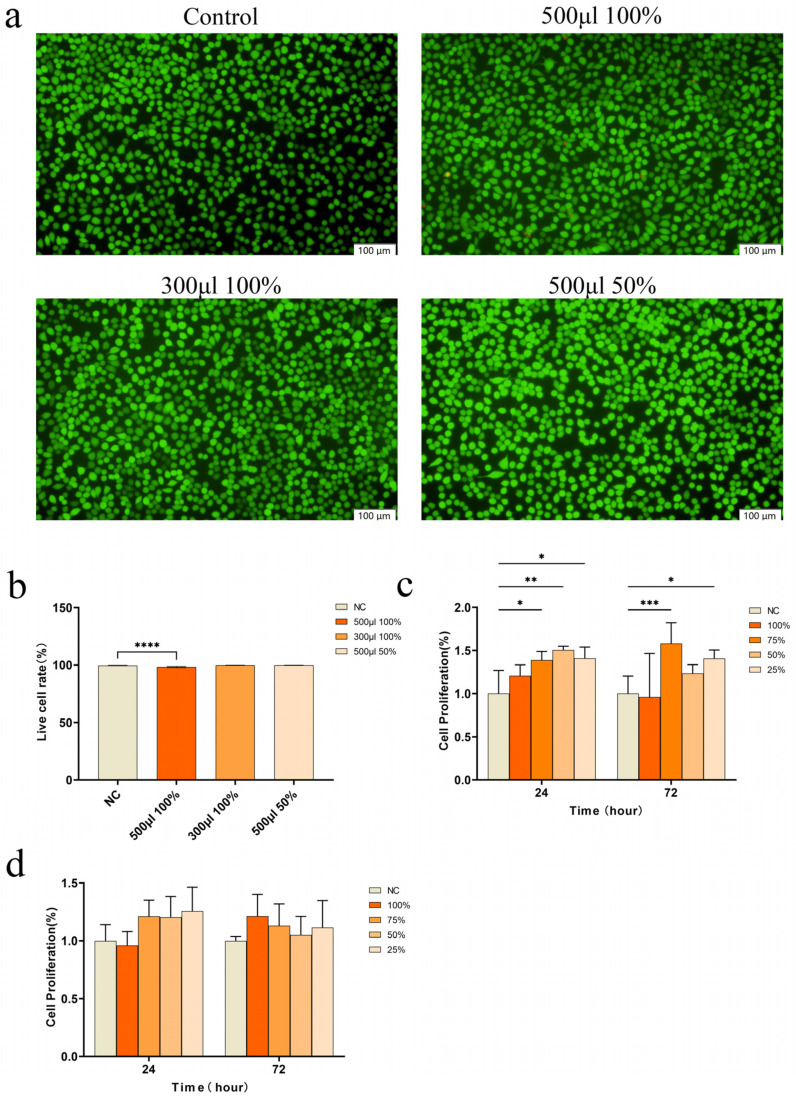
Cell experiments. (**a**) Live–dead cell staining; (**b**) Live cell rate analyzed by live–dead cell staining (**** *p* < 0.0001); (**c**) Cell proliferation of L929 co-cultured with different concentrations of CSMA/SI/AuNP hydrogel (* *p* < 0.05, ** *p* < 0.01 and *** *p* < 0.001); (**d**) Cell proliferation of HuEVCs co-cultured with different concentrations of CSMA/SI/AuNP.

Biocompatibility is a crucial determinant of biomaterials as it enables the survival, proliferation, and differentiation of cells at the injury site, thereby facilitating wound healing. We initially assessed cell viability using a live/dead cell staining assay (Figure 2a).

When cells were cultured with high-concentration hydrogel extract (500 μL of 100%), cell growth was observed to be healthy, with only a small number of dead cells. However, when cells were cultured with lower concentrations of hydrogel extract (300 μL of 100% extract or 500 μL of 50% extract), almost no dead cells were detected, and cell viability was maintained at approximately 99% across all experimental groups (Figure 2b). These results suggest that the hydrogel did not exhibit cytotoxicity. The cytotoxicity of the hydrogel was further assessed by evaluating its effects on the proliferation of L929 and HUEVC cells using a CCK-8 assay. The results (Figure 2c) indicated that CSMA/SI/AuNP hydrogels significantly enhanced the proliferation of L929 cells. In particular, the cell proliferation capacity was significantly enhanced after 72 h of co-culture with 75% of the hydrogel extracts compared to the control group (*p* < 0.001). But the hydrogels did not exert a substantial effect on HUEVC proliferation, and did not show any inhibitory effects (Figure 2d). These findings suggest that the CSMA/SI/AuNP hydrogel demonstrates good tissue compatibility, promotes fibroblast proliferation, and establishes a solid foundation for tissue repair.

### 3.6. Migration of HUEVCs Induced by CSMA/SI/AuNPs Hydrogels

**Figure 3:**
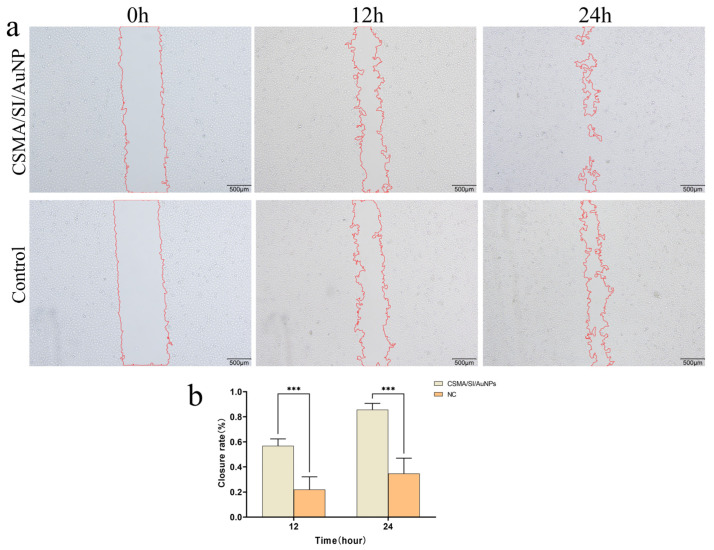
Cell experiments. (**a**) The Wound-Healing Assay of CSMA/SI/AuNP; (**b**) CSMA/SI/AuNP hydrogel cell scratch assay for closure rate (*** *p* < 0.001).

To explore whether the CSMA/SI/AuNP hydrogels influence angiogenesis, we conducted a scratch assay to examine their effects on HuVEC migration (Figure 3a,b). The results showed that the hydrogel significantly enhanced the wound closure rate compared to the control group (*p* < 0.001), indicating that CSMA/SI/AuNP hydrogels promote the migration of HuVECs. Previous studies demonstrated that gold nanoparticles (AuNPs) exhibit potent antiangiogenic effects by inhibiting endothelial cell proliferation in a dose-dependent manner in vitro, a mechanism that contributes to their antitumor properties [38,39]. However, in several studies related to tissue repair, AuNPs have not been shown to inhibit angiogenesis. Instead, biomaterials loaded with AuNPs may stimulate angiogenesis at the injury site [40,41]. These findings align with the results of the current study, where CSMA/SI/AuNP hydrogels did not inhibit HuVECs proliferation, instead significantly promoting HuVEC migration. This suggests that AuNP-loaded biomaterials contribute to angiogenesis, potentially providing a mechanism for enhancing tissue regeneration.

### 3.7. Acceleration of Diabetic Wound Healing by CSMA/SI/AuNP Hydrogels

**Figure 4:**
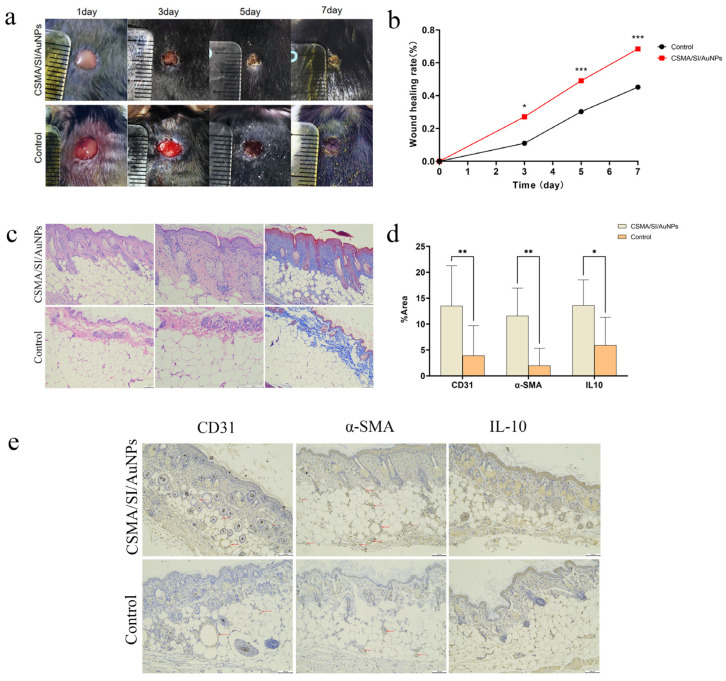
Animal experiments. (**a**) Digital photographs of wound healing on days 3, 5, and 7; (**b**) wound healing rate on days 3, 5, and 7 showed a statistical difference (*p* < 0.05) on day 3 and a statistically significant difference (*p* < 0.001) on days 5 and 7 (* *p* < 0.05 and *** *p* < 0.001); (**c**) H&E staining and Masson staining of injured skin tissue; (**d**) IHC quantitative analysis of various proteins revealed a statistically significant difference (*p* < 0.001) between the CD31 group and the IL-10 group, with data showing a statistical difference (*p* < 0.05) for the IL-10 group and a statistical difference (*p* < 0.01) for the α-SMA group (* *p* < 0.05 and ** *p* < 0.01); (**e**) IHC staining of injured skin tissue. The labeled points of CD31 and α-SMA groups were blood vessels.

To assess the effect of CSMA/SI/AuNP hydrogels on diabetic wound healing, a full-thickness skin defect model was established in diabetic mice, as depicted in Figure 4a. Over the 14-day in vivo study period, the wound closure rate in all experimental groups significantly increased on days 3, 5, and 7. As illustrated in Figure 4b, the CSMA/SI/AuNP hydrogel-treated group demonstrated significantly higher closure rates on days 3, 5, and 7 than the control group, with a marked difference on day 5 and day 7 (*p* < 0.001). On day 14, both the CSMA/SI/AuNP hydrogel and control groups showed near-complete wound healing, with no observable skin defects and no significant difference between the two groups. These results suggest that the CSMA/SI/AuNP hydrogels significantly accelerated the early stage wound healing process in diabetic mice.

The ultimate objective of clinical wound healing is not only the closure of the wound but also the restoration of the skin’s original functions, such as thermoregulation, sweat secretion, and barrier protection. An ideal wound dressing should promote rapid wound healing and the regeneration of skin appendages. To evaluate the effectiveness of the hydrogel in tissue repair, we performed H&E and Masson’s trichrome staining of wounded skin tissue (Figure 4c). Compared with the control group, the CSMA/SI/AuNP hydrogel group exhibited a more intact skin architecture, thicker collagen deposition, and more ordered collagen fiber arrangement. Furthermore, the control group exhibited significant inflammatory cell infiltration, including abundant neutrophils, particularly near the wound edges. In contrast, although inflammatory cell infiltration was still observed in the hydrogel-treated group, the density of these cells near the wound edge was reduced, suggesting that the hydrogel modulated the inflammatory response. Additionally, the CSMA/SI/AuNP hydrogel group displayed a greater number of skin appendages such as hair follicles and sweat glands, indicating that the hydrogel aids in the structural and functional restoration of damaged tissue.

### 3.8. CSMA/SI/AuNP Hydrogels Promote Angiogenesis and Modulate Inflammatory Responses

To further elucidate the effect of the hydrogel on angiogenesis and the inflammatory response, immunohistochemical staining was performed and analyzed for CD31, α-SMA, and IL-10 (Figure 4d,e). Compared with the control group, the CSMA/SI/AuNP hydrogel-treated group exhibited a significantly denser vascular network. Additionally, the expression of CD31 and α-SMA was markedly higher in the hydrogel group (*p* < 0.01), indicating that CSMA/SI/AuNP hydrogels promote angiogenesis, thereby facilitating wound healing in diabetic conditions. Based on cellular experiments, our findings indicate that CSMA/SI/AuNP hydrogels significantly enhance the migration of vascular endothelial cells without inhibiting their proliferation. In conjunction with in vivo experiments, we hypothesize that this hydrogel promotes angiogenesis by enhancing the migratory capacity of injured peripheral vascular endothelial cells while maintaining their proliferative potential.

We also observed that the expression of IL-10 was significantly elevated in the hydrogel-treated group (*p* < 0.05), implying that the hydrogel modulated the local immune response by promoting the secretion of the anti-inflammatory cytokine IL-10. This could reduce excessive inflammation and accelerate the healing process. These findings are consistent with those of previous studies that demonstrated the beneficial effects of soybean isoflavones, a key component of the hydrogel, in promoting angiogenesis and modulating inflammatory response. For example, in a study by Zhang et al. (2020), a soybean protein solution (SPS) accelerated wound healing, stimulated collagen deposition, and promoted the migration of human umbilical epithelial stem cells (HUESCs) in a dose-dependent manner. Furthermore, soybean isoflavones were found to reduce reactive oxygen species (ROS) and induce macrophage polarization towards the M2 phenotype, thereby enhancing angiogenesis and mitigating inflammatory response [42]. Our results support the role of soy isoflavones in promoting angiogenesis and modulating inflammation.

## 4. Limitation

Although this study has demonstrated that CSMA/SI/AuNP hydrogels facilitate wound healing in diabetic conditions, the specific molecular mechanisms responsible for this therapeutic effect remain inadequately explored. Additionally, the long-term consequences of CSMA/SI/AuNP hydrogel application on the injured tissue, such as potential scarring or fibrosis, have not been thoroughly investigated. Further research is needed to elucidate the underlying biological processes and assess the enduring impact of hydrogel treatment on tissue regeneration and integrity.

## 5. Conclusions

This study developed a novel hydrogel composed of chitosan methacryloyl (CSMA), soy isoflavones (SIs), and gold nanoparticles (AuNPs). The results demonstrated that the hydrogel possesses excellent histocompatibility and significantly enhances tissue repair by promoting collagen deposition, facilitating angiogenesis, suppressing local inflammation, accelerating wound healing, and supporting the regeneration of skin appendages. As a light-responsive hydrogel that is injectable, it fills wounds perfectly and has good mechanical properties after a simple stimulation. Additionally, it is non-toxic, making it ideal for treating complex diabetic or chronic wounds. This hydrogel is an effective and versatile biomaterial that offers rapid restoration of both the structural and functional properties of damaged skin, providing an ideal dressing for clinical wound management.

## Data Availability

The data used to support the findings of this study are available from the corresponding author upon request.

## Abbreviations

The following abbreviations are used in this manuscript:

CSMA	Chitosan Methacryloyl
SIs	Soy isoflavones
AuNP	Gold nanoparticle
HuVECs	Human umbilical vein endothelial cell
ROS	Reactive oxygen species
CS	Chitosan
MA	Methacrylic acid
UV	Ultraviolet
TEM	Transmission electron microscopy
FT-IR	Fourier transform infrared spectroscopy
PBS	Phosphate-buffered saline
CO_2_	Carbon dioxide
PI	Propidium iodide
FBS	Fetal bovine serum
RPMI	Roswell Park Memorial Institute
EDTA	Ethylene Diamine Tetraacetic Acid
α-SMA	α-Smooth Muscle Actin
IL-10	Interleukin-10
